# Fatal outcome of SARS-CoV-2 infection (B1.1.7) in a 4-year-old child

**DOI:** 10.1007/s00414-021-02687-9

**Published:** 2021-09-12

**Authors:** Johanna Menger, Sofia Apostolidou, Carolin Edler, Inga Kniep, Robin Kobbe, Dominique Singer, Jan-Peter Sperhake

**Affiliations:** 1 Department of Legal Medicine, University Medical Center Hamburg-Eppendorf, Butenfeld 34, 22529 Hamburg, Germany; 2 Department of Neonatology and Pediatric Intensive Care Medicine, University Medical Center Hamburg-Eppendorf, Martinistr. 52, 20246 Hamburg, Germany; 3 First Department of Medicine, Division of Infectious Diseases, University Medical Center Hamburg-Eppendorf, Martinistr. 52, 20246 Hamburg, Germany

**Keywords:** COVID, SARS-CoV-2, B1.1.7, Autopsy, Children, MIS-C

## Abstract

This case report highlights details of a case of critical acute respiratory syndrome coronavirus type 2 (SARS-CoV-2) with B1.1.7 variant in a 4-year-old girl who died due to pneumonia and pulmonary hemorrhage. The girl was referred to our University ECMO Center from another University hospital for veno-arterial extracorporeal membrane oxygenation (VA-ECMO). In the clinical course, superinfection with *Pseudomonas aeruginosa* was detected. Virological evidence of herpes simplex sepsis was also obtained in blood samples on her day of death. Transcription polymerase chain reaction (PCR) confirmed SARS-CoV-2 infection in lung tissue. Postmortem computed tomography showed pulmonary hemorrhage with inhomogeneous density values in both lungs. Lung tissue showed no ventilated areas. Autopsy revealed a massively congested lung with evidence of acute respiratory distress syndrome (ARDS) and pneumonia with multiple abscesses. Histopathology showed a mixture of diffuse alveolar injury with hyaline membranes, massive hemorrhage, and bronchopneumonia with multiple granulocytic abscesses. Cardiac examination revealed pericarditis. Suspicion of myocarditis or myocardial infarction could not be confirmed microscopically. To our knowledge, this is the first autopsy-based case report of the death of a previously healthy child due to the new variant B 1.1.7 in Germany.

**Supplementary Information:**

The online version contains supplementary material available at 10.1007/s00414-021-02687-9.

## Introduction

According to clinical experience and studies to date, the course of SARS-CoV-2 infection in children is less severe than in adults, often asymptomatic or mild, so the prognosis is considered good in general [3, 13, 20]. There have been few reports of severe clinical courses in children associated with SARS-CoV-2 infection, although fatal COVID-19 remains a rarity. In most of these cases, the children suffered from a condition called multisystemic inflammatory syndrome in children (MIS-C) [1, 2, 10]. MIS-C associated with SARS-CoV-2 infection resembles Kawasaki disease but has distinctive features in clinical presentation [19]. Clinical criteria for diagnosis have been defined by various organizations such as WHO [25] and the Royal College of Paediatrics and Child Health (RCPCH) [22].

Since the new variant B 1.1.7 first appeared in Germany in December 2020, questions have been raised about whether this new lineage can also lead to more severe clinical courses in children [8]. Fatal cases in children were reported to be higher during periods of higher infection rates in the community, but a general raise in mortality over time has not been observed [6, 7, 12]. However, the clinical impact of the new SARS-CoV-2 variant B.1.1.7 on children is not yet fully understood. Overall, there is little autopsy data in children with COVID-19 and MIS-C with and without variant B1.1.7. This case report details one of these critical cases in a 4-year-old girl.

## Case-report

A 4-year-old girl died in the first quarter of 2021 at a University Medical Center due to massive pulmonary hemorrhage with low cardiac output in superinfected pneumonia caused by SARS-CoV-2 variant B.1.1.7.

She was referred to our pediatric intensive care unit (PICU) after being treated at another hospital for 7 days. Apart from obesity (BMI 21.7 kg/m^2^; > 99th percentile), she had no other previous medical conditions.

She was originally presented to a general pediatrician for respiratory distress. Suspecting acute laryngotracheitis, she was temporarily treated orally with dexamethasone and cough suppressant. As her symptoms worsened that same day, she was taken to a University Emergency Department. On admission to the pediatric ward (day 1), she had abdominal pain, inspiratory stridor, papules at the corners of her mouth, cough, blood oxygen saturation of 97%, heart rate of 175 bpm, and hypertension (132/102 mmHg). Fever was not detected. Family history did not reveal any predisposition to arterial hypertension.

On day 2, the SARS-CoV-2 PCR on the nasopharyngeal swab was positive. Due to rising C-reactive protein (CRP) levels, the previous therapeutic regimen was extended and antibiotic therapy (ampicillin/sulbactam) was started. Initially, noninvasive ventilation with high flow/NIV was attempted. However, with progressive global respiratory failure (hypoxic and hypercapnic), intubation was carried out on the same day, and she was mechanically ventilated for 2 days.

Chest radiographs on day 4 and day 6 showed pulmonary opacification that began in the right upper lobe and then spread to the entire right lung and left lower lobe. At this time, there was no microbiological evidence of bacterial superinfection. After presumed stabilization, extubation was performed on day 6. However, on the same evening, there was an increasing deterioration and a rather rapid reintubation with now significantly worse overall situation. The child was barely ventilable, with increasing circulatory insufficiency and lactic acidosis. During day 7, the girl could only be ventilated on the bag valve mask (BVM). She received high doses of catecholamines. When she was no longer fit for transport, emergency cannulation for ECMO was performed before transfer to the university hospital.

A bronchoscopy (day 10) showed macroscopically thick bloody mucus. Typical findings of ARDS such as hyaline membranes were found in the lung biopsies taken.

Furthermore, the SARS-CoV-2 variant of concern (VOC) B.1.1.7 was detected [16], and it was confirmed that other family members also tested positive for B.1.1.7.

Initially, a very high viral load was detected in blood (380,000 copies/ml) and tracheal secretions (15 billion copies/ml), but after antiviral therapy with remdesivir (5 mg/kg, followed by 2.5 mg/kg for 8 days) and a single dose of the monoclonal antibody bamlanivimab 700 mg, SARS-CoV-2-RNA copies/ml gradually declined. Based on the clinical and laboratory changes, antibiotic therapy was altered, and eventually pseudomonads were detected in the tracheal secretions. Furthermore, herpes simplex virus (HSV-1; 540,000 copies/ml plasma) viremia was detected from a blood sample obtained when the child’s condition, especially with regard to ventilation, critically deteriorated. On day 22, after 17 days on ECMO, the patient died of severe pulmonary failure with pulmonary hemorrhage, ECMO arrest, and cardiac failure.

**Fig. 1:**
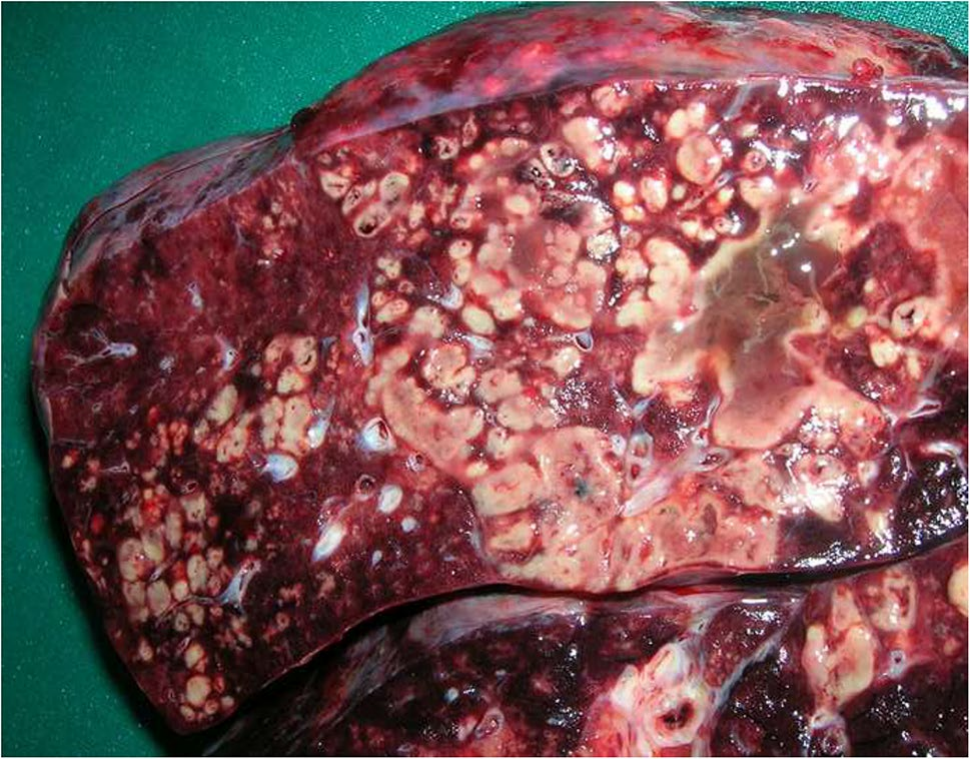
Cutting surface of the lung with massive abscessed pneumonia

*Postmortem computed tomography* showed pulmonary hemorrhage with inhomogeneous consolidations in both lungs (Fig. 1 – electronic supplementary material), a small hematothorax bilaterally with regularly inserted drains, and a small pericardial effusion. No ventilated areas in the lungs could be delineated.

### Autopsy findings

The lungs were massively congested, firm, and heavy (right: 513 g; left: 442 g). The lung surfaces showed fibrinous purulent pleurisy (Fig. 2 – electronic supplementary material). There were multiple regions of multifocal hemorrhage. Both lungs showed massive abscessed pneumonia (Fig. 1). The bronchi were almost completely congested with viscous mucus. The pericardium showed similar fibrin deposits consistent with pericarditis.

The posterior wall of the heart showed small-spot fading, so that myocarditis or a recent infarction was initially suspected. In addition, there were findings consistent with multi-organ failure (e.g., cerebral edema) and a splenic infarct measuring 2 × 2 × 0.5 cm.

The liver showed a patchy, autumn leaf-like appearance consistent with shock liver. There was no evidence of previously unrecognized preexisting internal diseases.

### Postmortem microbiology and virology

Transcription polymerase chain reaction (PCR) confirmed SARS-CoV-2 infection in the lungs. Superinfection with *Pseudomonas aeruginosa* in lung tissue and HSV-1 viremia blood samples (260,000 copies/ml) was confirmed postmortem.

### Histopathological features

We performed routine hematoxylin–eosin staining for histopathologic evaluation. The lungs showed severe damage with a mixture of massive hemorrhage with blood pooling in almost all alveoli, bronchopneumonia (Figs. 4 and 5 – electronic supplementary material), and diffuse alveolar damage (DAD) with the presence of hyaline membranes (Fig. 6– electronic supplementary material). Large abscesses occurred in multiple areas of both lungs, as is typically the case with bacterial sepsis. Furthermore, the upper airways showed moderate tracheobronchitis characterized by lympho-plasma cellular inflammation. Macroscopically suspected myocarditis could not be confirmed. The heart showed no pathological changes except for fibrinous pericarditis (Fig. 7 – electronic supplementary material). The other organs were consistent with age.

## Discussion

Since the onset of the SARS-CoV-2 pandemic in the first quarter of 2020, forensic medicine has played an integral role in the understanding of the disease. The first autopsy of a COVID-19 death from Wuhan, China, was reported in the Chinese Journal of Forensic Medicine back in February 2020 [14]. What remains the largest consecutive autopsy series involving 80 COVID-19 decedents has been published in the International Journal of Legal Medicine [11]. The importance of forensic medicine as an investigator not only in criminal proceedings but also in public health protection issues has been emphasized in the context of the SARS-CoV-2 pandemic [18, 21, 23]. This shows that in many countries, forensic medicine has taken over tasks that were previously performed only by clinical pathology. In Hamburg, virtually all autopsies on COVID-19 decedents were performed by the Department of Legal Medicine on behalf of the health authorities.

Although severe and critical SARS-CoV-2-associated disease has been described for MIS-C, COVID-19 mortality is usually low in children [10, 12]. The case presented here is one of the rare fatal courses of pediatric COVID-19 with ARDS and superinfection.

Autopsy findings of the lungs showed bronchopneumonia with multiple abscesses. Furthermore, both lungs showed diffuse consolidation changes, edema, and massive hemorrhage. Microscopically, typical signs of DAD were present, similar to those found in adult decedents with COVID-19. This is consistent with the results of biopsies taken antemortem. Most likely, the DAD in this child was also due to infection with SARS-CoV-2, whereas the abscesses were likely caused by superinfection with *Pseudomonas aeruginosa*.

There are various reports of cardiac failure due to MIS-C in children Myocarditis and involvement of the coronaries are also described [4, 5, 9, 19], which were not detectable in the autopsy. Even though myocarditis was first suspected, it could also not be confirmed histologically. However, this does not rule out very recent damage to the myocardium, which may not yet have been demarked.

The lungs were congested with blood and heavy. This is consistent with autopsy findings from other cases [24]. Particularly striking was the high weight of the lungs (right: 513 g; left 442 g) in relation to age and body length (age-related norm: right lung: 60–306 g; left lung: 54–270 g; height class reference: right lung: 51–337 g; left lung 42–303 g) [15]. In addition to the massive inflammatory changes in the lungs, the girl’s obesity (BMI > 99th percentile) was likely a contributing factor to the fatal course of the disease. In her case, all organs except the spleen had weights that were above average for her age. Other published case reports and studies suggest that obesity increases the risk for severe courses of SARS-CoV-2 infection in adults and children [2, 4, 17].

As of April 2021, there have been only two cases of fatal SARS-CoV-2 infection in children at the University Medical Center Hamburg Eppendorf, and only that case was variant B.1.1.7.

## Supplementary Information

Below is the link to the electronic supplementary material.

Fig. 1 electronic supplementary material: Postmortem-CT showing consolidations in both lungs. Note the unventilated, solid nature of the lung tissue in the "lung window"! (JPG 46.7 KB)

Fig. 2 electronic supplementary material: Lung surface with fibrinous purulent pleurisy (JPG 2.43 MB)

Fig. (JPG 1.86 MB)

Fig. 4 – electronic supplementary material: Purulent abscessed bronchopneumonia with congestion and dense leucocytic infiltrate (Hematoxilin and Eosine, x50). (JPG 3.73 MB)

Fig. 5 – electronic supplementary material: Purulent bronchitis (Hematoxilin and Eosine, x80). (JPG 348 MB)

Fig. 6 – electronic supplementary material: Hyaline membranes and blood congestion with a mixed intraalveolar infiltrate (Hematoxilin and Eosine, x100) (JPG 3.21 MB)

Fig. 7 – electronic supplementary material: Fibrinous pericarditis (Hematoxilin and Eosine, x80). (JPG 3.20 MB)
