# Supplementary figures and images for: Fatal outcome of SARS-CoV-2 infection (B1.1.7) in a 4-year-old child
Source: Int J Legal Med. 2021 Sep 12;136(1):189–92. doi: 10.1007/s00414-021-02687-9 (PMC8435154; doi:10.1007/s00414-021-02687-9)

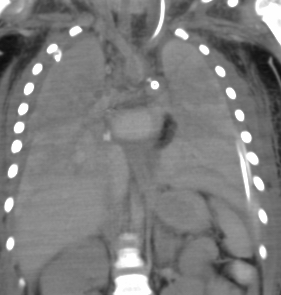

Supplement: Supplementary file 1 — Fig. 1 electronic supplementary material: Postmortem-CT showing consolidations in both lungs. Note the unventilated, solid nature of the lung tissue in the "lung window"! (JPG 46.7 KB) [file 414_2021_2687_MOESM1_ESM.jpg]

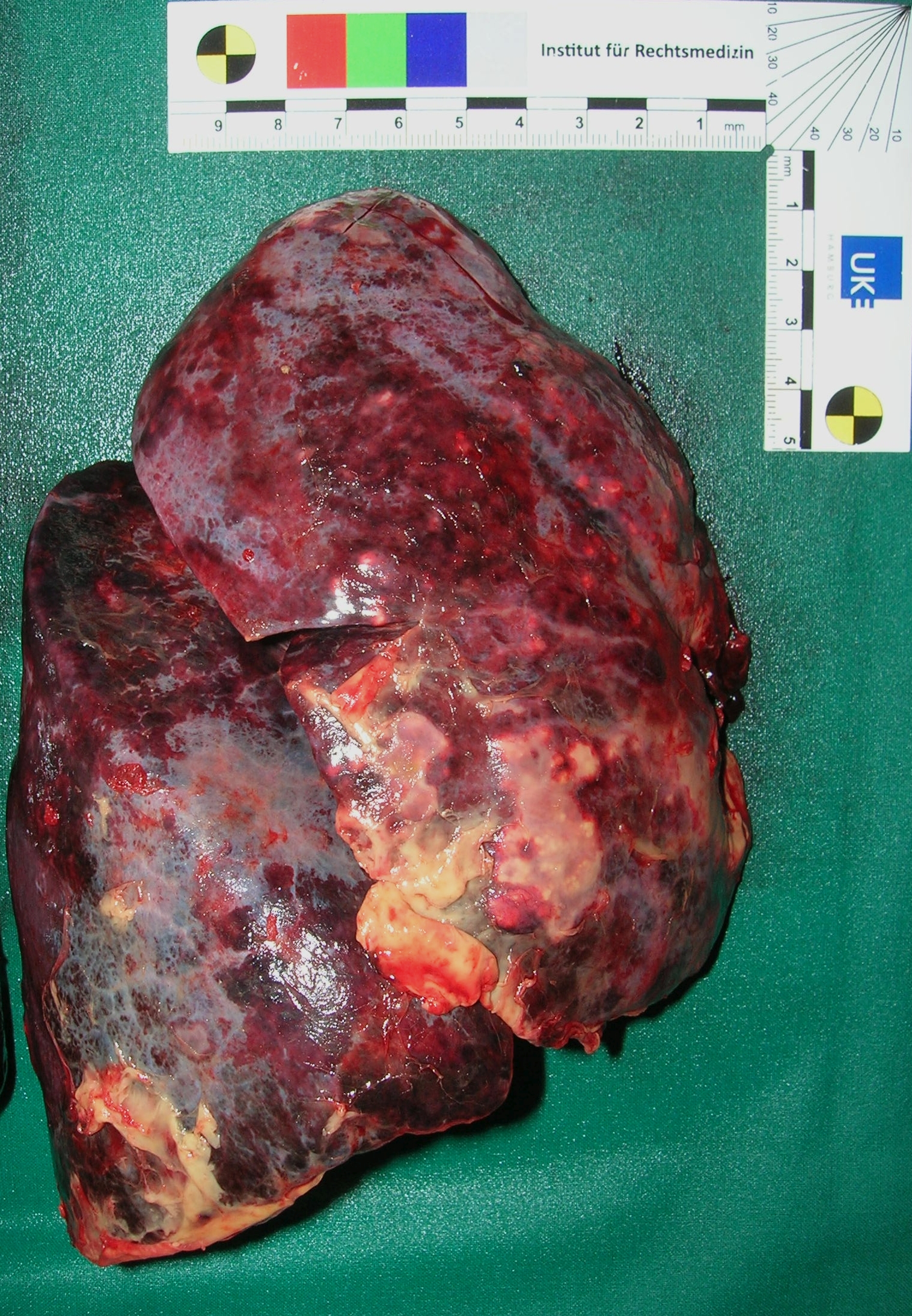

Supplement: Supplementary file 2 — Fig. 2 electronic supplementary material: Lung surface with fibrinous purulent pleurisy (JPG 2.43 MB) [file 414_2021_2687_MOESM2_ESM.jpg]

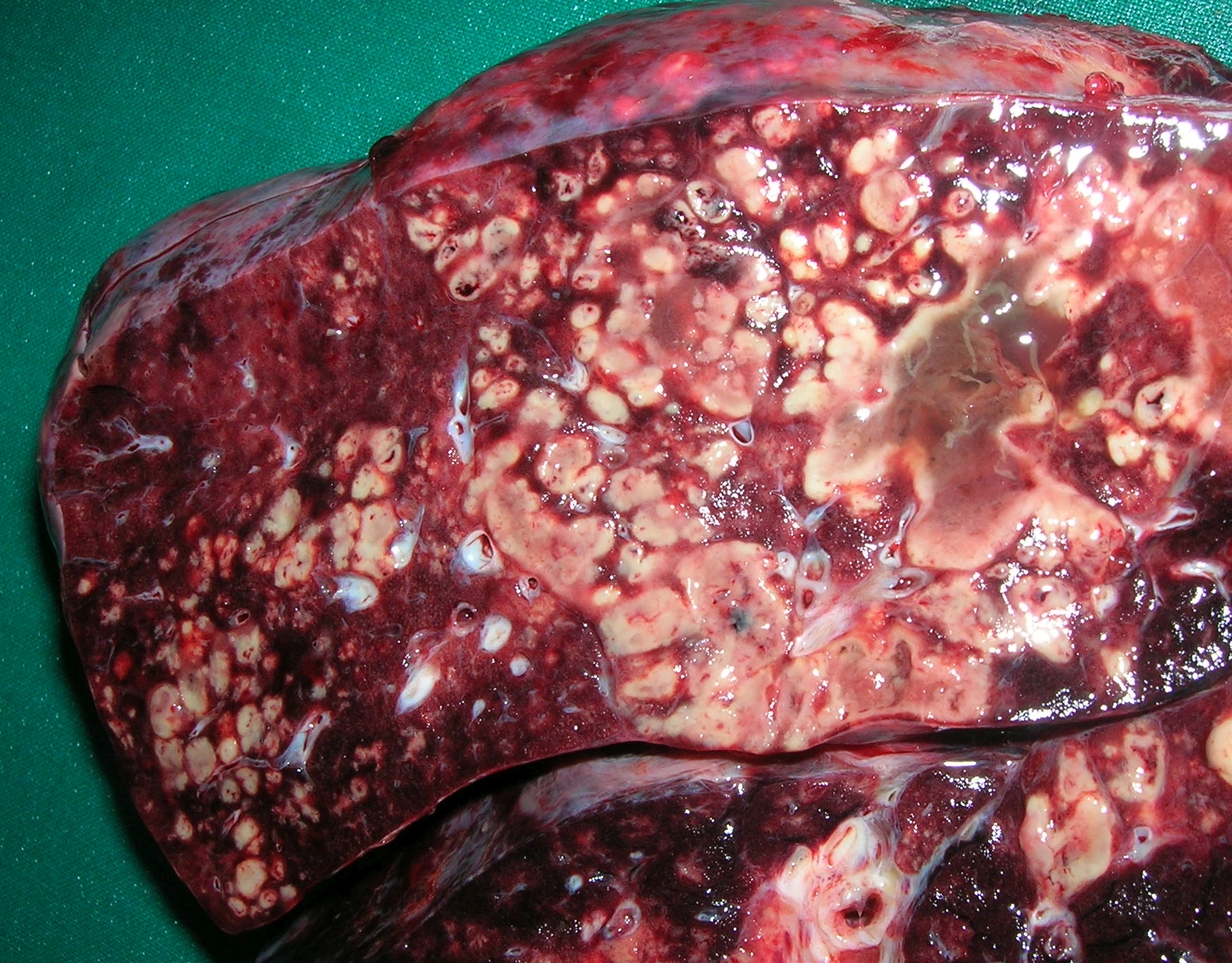

Supplement: Supplementary file 3 — Fig. (JPG 1.86 MB) [file 414_2021_2687_MOESM3_ESM.jpg]

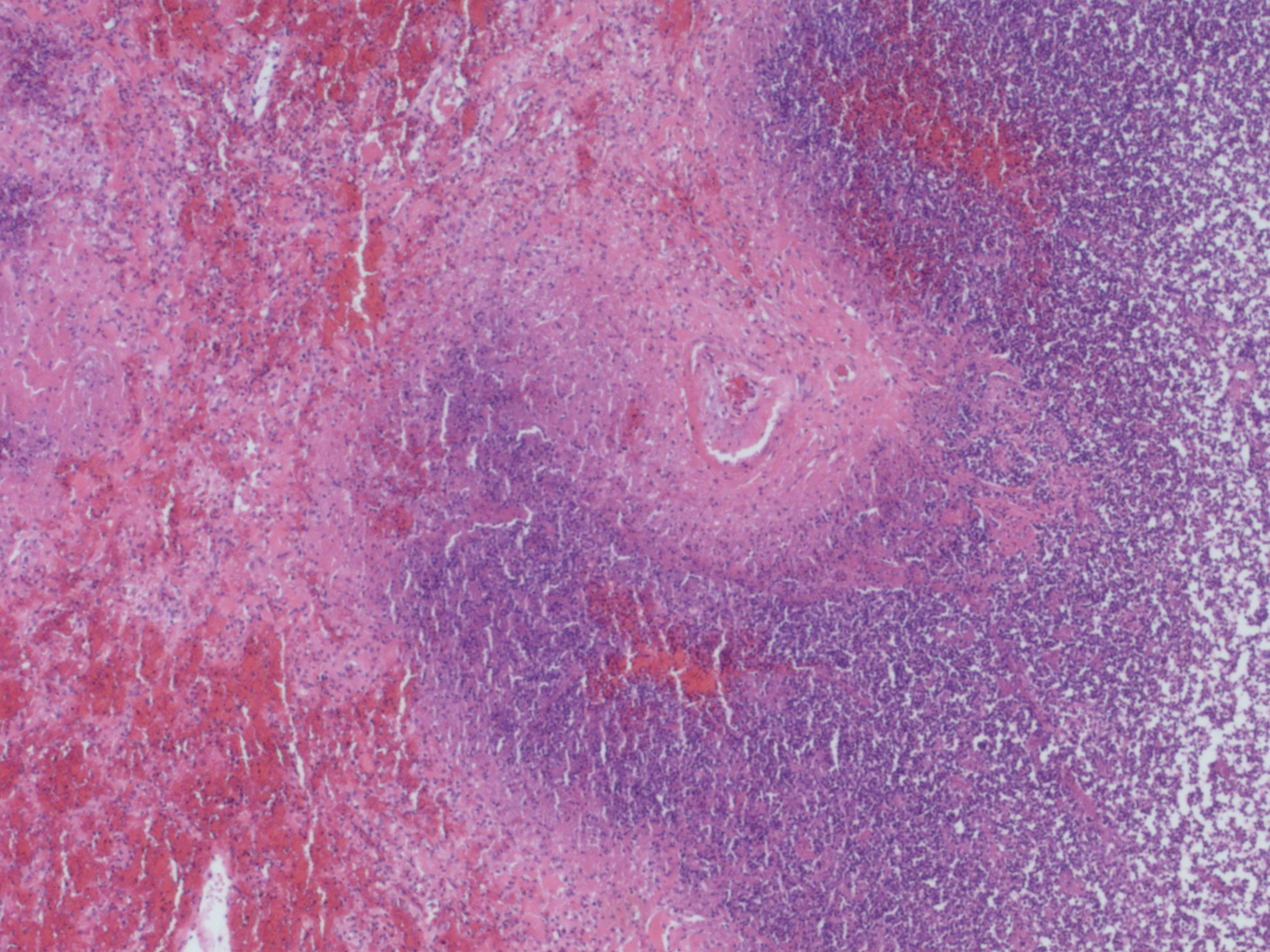

Supplement: Supplementary file 4 — Fig. 4 – electronic supplementary material: Purulent abscessed bronchopneumonia with congestion and dense leucocytic infiltrate (Hematoxilin and Eosine, x50). (JPG 3.73 MB) [file 414_2021_2687_MOESM4_ESM.jpg]

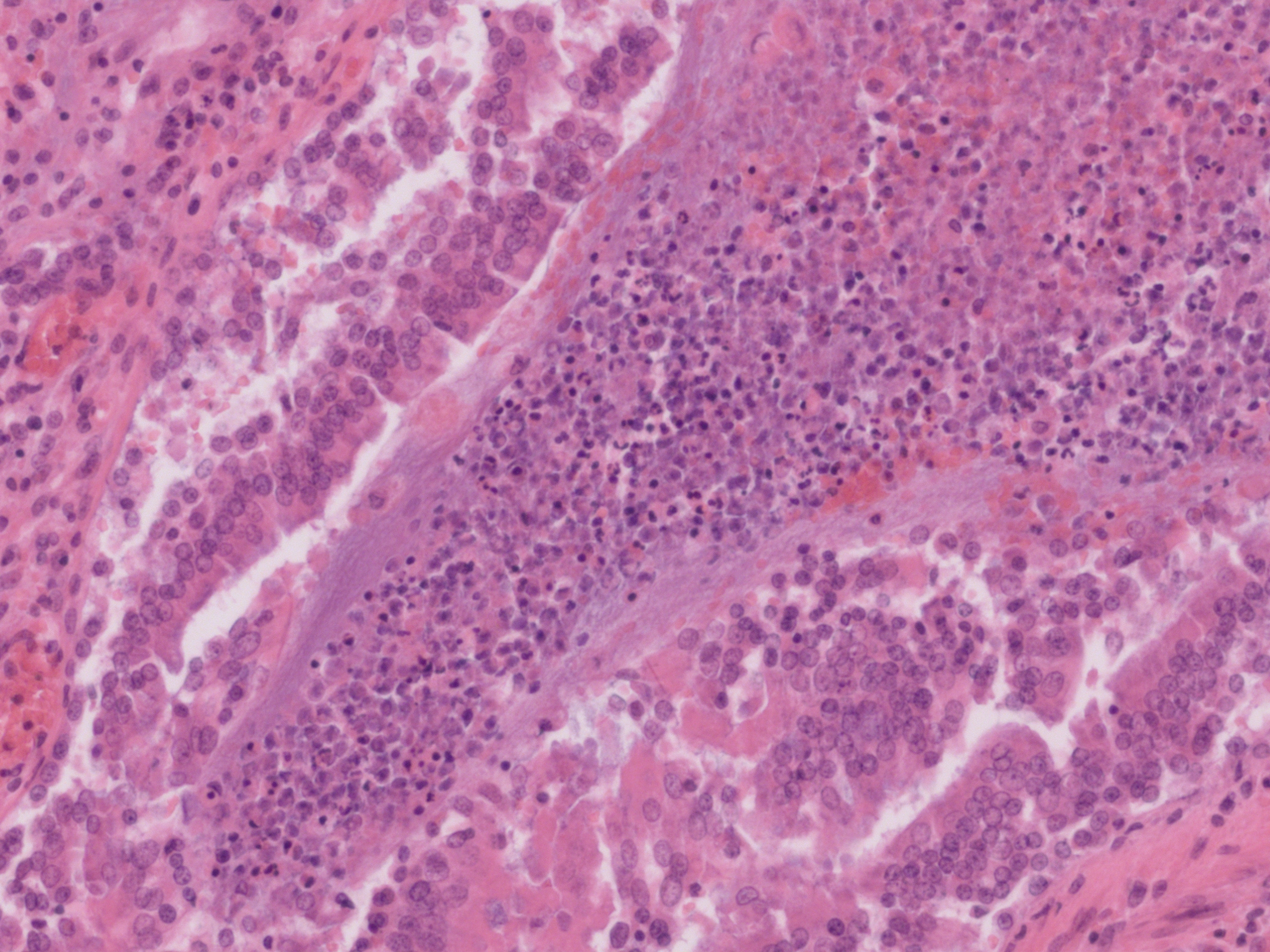

Supplement: Supplementary file 5 — Fig. 5 – electronic supplementary material: Purulent bronchitis (Hematoxilin and Eosine, x80). (JPG 348 MB) [file 414_2021_2687_MOESM5_ESM.jpg]

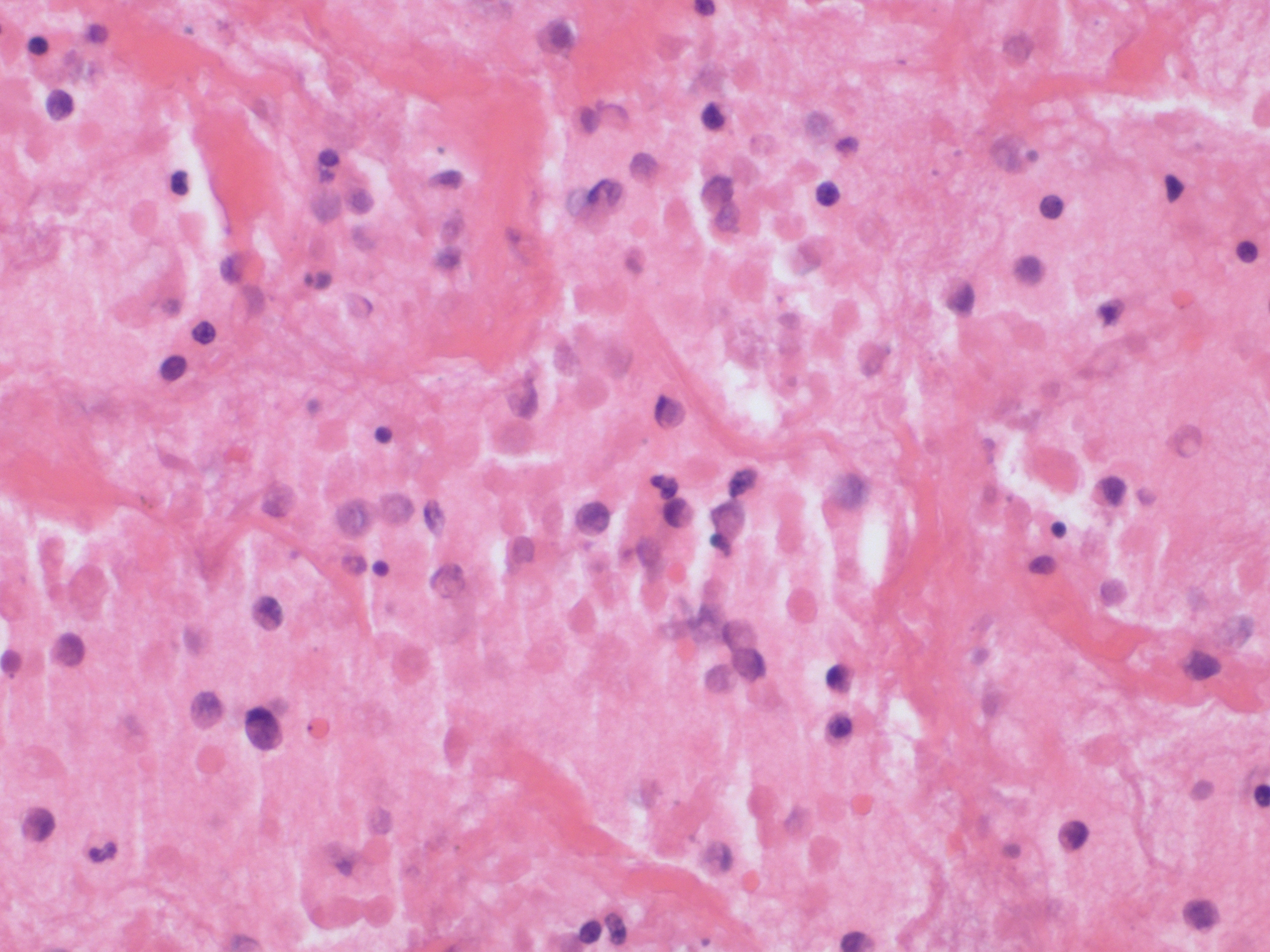

Supplement: Supplementary file 6 — Fig. 6 – electronic supplementary material: Hyaline membranes and blood congestion with a mixed intraalveolar infiltrate (Hematoxilin and Eosine, x100) (JPG 3.21 MB) [file 414_2021_2687_MOESM6_ESM.jpg]

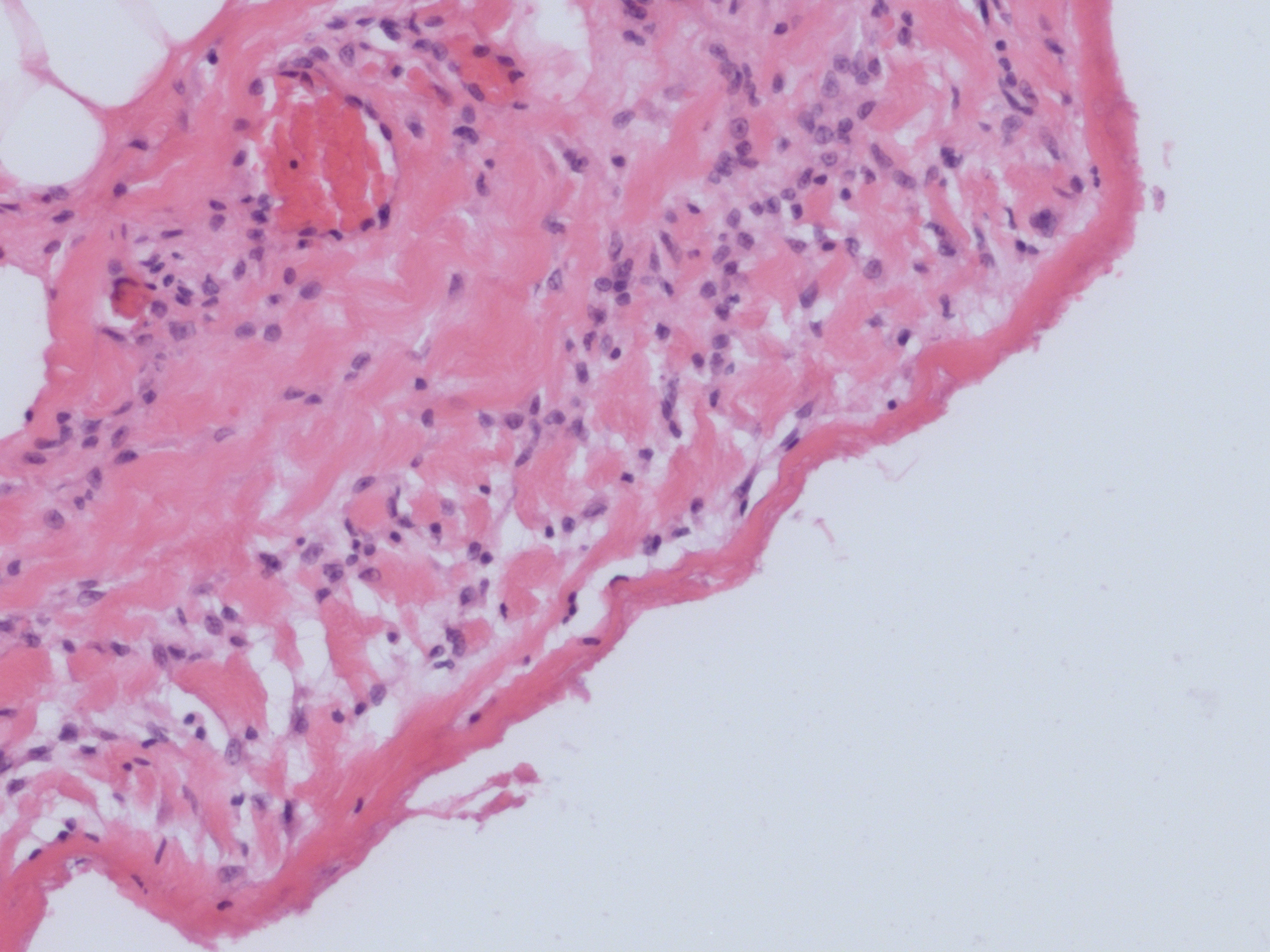

Supplement: Supplementary file 7 — Fig. 7 – electronic supplementary material: Fibrinous pericarditis (Hematoxilin and Eosine, x80). (JPG 3.20 MB) [file 414_2021_2687_MOESM7_ESM.jpg]
